# Role of Diatoms in the Spatial-Temporal Distribution of Intracellular Nitrate in Intertidal Sediment

**DOI:** 10.1371/journal.pone.0073257

**Published:** 2013-09-04

**Authors:** Peter Stief, Anja Kamp, Dirk de Beer

**Affiliations:** 1 Max Planck Institute for Marine Microbiology, Microsensor Group, Bremen, Germany; 2 University of Southern Denmark, Institute of Biology, NordCEE, Odense, Denmark; 3 Jacobs University Bremen, Molecular Life Science Research Center, Bremen, Germany; National Institute of Water & Atmospheric Research, New Zealand

## Abstract

Intracellular nitrate storage allows microorganisms to survive fluctuating nutrient availability and anoxic conditions in aquatic ecosystems. Here we show that diatoms, ubiquitous and highly abundant microalgae, represent major cellular reservoirs of nitrate in an intertidal flat of the German Wadden Sea and are potentially involved in anaerobic nitrate respiration. Intracellular nitrate (ICNO_3_) was present year-round in the sediment and was spatially and temporally correlated with fucoxanthin, the marker photopigment of diatoms. Pyrosequencing of SSU rRNA genes of all domains of life confirmed that ICNO_3_ storage was most likely due to diatoms rather than other known nitrate-storing microorganisms (i.e., large sulfur bacteria and the eukaryotic foraminifers and gromiids). Sedimentary ICNO_3_ concentrations reached up to 22.3 µmol dm^-3^ at the sediment surface and decreased with sediment depth to negligible concentrations below 5 cm. Similarly, the ICNO_3_/fucoxanthin ratio and porewater nitrate (PWNO_3_) concentrations decreased with sediment depth, suggesting that ICNO_3_ of diatoms is in equilibrium with PWNO_3_, but is enriched relative to PWNO_3_ by 2-3 orders of magnitude. Cell-volume-specific ICNO_3_ concentrations in a diatom mat covering the sediment surface during spring were estimated at 9.3-46.7 mmol L^-1^. Retrieval of 18S rRNA gene sequences related to known nitrate-storing and nitrate-ammonifying diatom species suggested that diatoms in dark and anoxic sediment layers might be involved in anaerobic nitrate respiration. Due to the widespread dominance of diatoms in microphytobenthos, the total nitrate pool in coastal marine sediments may generally be at least two times larger than derived from porewater measurements and partially be recycled to ammonium.

## Introduction

In aquatic microbial communities, nitrate serves both as a nitrogen source for assimilation, and as an electron acceptor for anaerobic respiration. Nitrate availability is thus a key environmental factor controlling primary production and anaerobic energy production in many aquatic ecosystems. Microbial primary producers are the major assimilators of nitrate in euphotic layers of the water column and sediments [1,2]. Microorganisms capable of anaerobic nitrate reduction (i.e., denitrification, dissimilatory nitrate reduction to ammonium (DNRA), and indirectly anaerobic ammonium oxidation (anammox)) are the major consumers of nitrate in anoxic water and sediment layers [3]. For both assimilatory and dissimilatory use of nitrate, microorganisms evolved physiological mechanisms to secure nitrate supply under nitrate-limited or nitrate-fluctuating conditions, such as high-affinity nitrate transporters [4] and liquid vacuoles for ICNO_3_ storage [5].

ICNO_3_ storage occurs in both prokaryotic and eukaryotic microorganisms. The large sulfur bacteria *Beggiatoa* , *Thioploca* , and *Thiomargarita* (all belonging to the family Thiotrichaceae within the γ-Proteobacteria) possess vacuoles in which nitrate is stored at concentrations reaching several hundred millimolar [6–10]. Large sulfur bacteria often occur at extremely high abundance in sulfidic habitats of aquatic ecosystems. The large nitrate stores are used to oxidize sulfide in the absence of oxygen, commonly with ammonium as the end product of nitrate reduction [11]. More recently, vacuolar ICNO_3_ storage of up to a few hundred millimolar was discovered in Foraminifera and Gromiida [12–15]. These eukaryotic organisms (and possibly their endosymbionts) use their nitrate stores for denitrification to dinitrogen or nitrous oxide, which allows them to survive in anoxic sediment layers. Just like the large sulfur bacteria, Foraminifera and Gromiida are able to migrate between nitrate-containing and nitrate-free layers in the sediment.

ICNO_3_ storage is also widespread in the eukaryotic phytoplankton of the world’s oceans. Pelagic diatoms have long been recognized to accumulate nitrate intracellularly and use it for nitrogen assimilation [16]. Under laboratory conditions (i.e., with nitrate in the medium), pelagic diatoms have been found to store nitrate up to 200 mmol L^-1^ [17–20]. A limited number of other phytoplankton species belonging to the phyla Haptophyta, Dinoflagellata, and Chlorophyta were also shown to store nitrate intracellularly at concentrations lower than 15 mmol L^-1^ [17,18]. However, given their vast abundance, pelagic diatoms might represent the largest cellular reservoirs of nitrate in the world’s oceans.

Benthic microalgae storing nitrate have received much less attention. Maximum ICNO_3_ concentrations of 274 mmol L^-1^ [20] and 447 mmol L^-1^ [21] were measured in isolated benthic diatoms, however after pre-incubation with nitrate. Notably, an axenic strain of the benthic diatom *Amphora* *coffeaeformis* reduced its ICNO_3_ to ammonium when incubated under dark, anoxic conditions that prevail in deeper sediment layers [20]. This metabolic pathway of anaerobic energy production is primarily found in Bacteria and Archaea and rarely in Eukarya. *In situ* ICNO_3_ concentrations of benthic diatoms have not been reported previously, but the occurrence of benthic ICNO_3_ has sometimes been discussed to originate from diatoms [10,22–25]. Diatoms dominate the microphytobenthos of intertidal sediments [26–29] and settle in masses onto coastal bay sediments following seasonal phytoplankton blooms [22]. For these reasons, coastal marine sediments might be important sites for ICNO_3_ storage by diatoms. This potentially large nitrate pool has implications for nitrogen assimilation and anaerobic nitrate reduction by the diatoms themselves, but also for anaerobic nitrate reduction by the microbial sediment community when diatoms are mineralized [30].

In this study, depth distribution and seasonal changes of the ICNO_3_ pool of intertidal sediment in the German Wadden Sea were analyzed along with the water column and porewater pools of nitrate. The ICNO_3_ pool was directly linked to the occurrence of diatoms by measuring sedimentary fucoxanthin and chlorophyll *a* concentrations. The benthic community was screened for known nitrate-storing microorganisms by pyrosequencing of SSU rDNA genes of all domains of life. The possibility of *in situ* dissimilatory nitrate reduction by diatoms was assessed from the occurrence of known nitrate-respiring diatom species. A diatom mat established on the sediment surface during spring received particular attention in terms of community analysis and estimation of cell-specific ICNO_3_ concentrations.

## Materials and Methods

### Study site and sample collection

An intertidal flat in the German Wadden Sea near Dorum-Neufeld (53°45'N, 8°21'E) which is open to the public for recreational activities was investigated in 2011 and 2012. The permission to collect sediment samples for scientific purposes was issued by the Nationalparkverwaltung Niedersächsisches Wattenmeer (Wilhelmshaven, Germany). The sampling campaigns did not involve endangered or protected species. The sandy-to-silty sediment of the intertidal flat was repeatedly sampled at low tide and between 9 and 11 a.m. using acrylic core liners (inner diameter 3.6 cm, length 18 cm). Replicate sediment cores were taken at randomly chosen spots within a sampling plot of 25×25 m that was located approximately 100 m away from the high-water line. Macrofaunal burrows were avoided during sampling. The freshly retrieved sediment cores were 12-13 cm long and were overlain by 0.5-1 cm of seawater. Slicing of sediment cores was carried out directly in the field with a portable core extruder. Surface water was collected in 3-5 ca. 5 cm-deep puddles on the sampling plot. Based on interfacial nitrate fluxes derived from microsensor profiles [59], the maximum change in nitrate concentration in these puddles was < 0.5 µmol L^-1^ during the time elapsed since high tide (maximally 2 h). Nitrate concentrations in these puddles can thus be considered representative of nitrate concentrations in the water column at high tide. Temperature was measured with a needle probe at a sediment depth of 5 cm. Sediment and water samples were stored on ice and processed in the laboratory within 1.5 h.

### Study design

The role of diatoms in ICNO_3_ storage in intertidal sediment was assessed using three different approaches:

1 The sedimentary contents of porewater and intracellular nitrate as well as the sedimentary contents of photopigments were measured in monthly intervals in 2011.
2 The diversity of prokaryotic and eukaryotic microorganisms potentially involved in ICNO_3_ storage was studied in June 2011 and December 2011 when ICNO_3_ contents had reached the lowest and highest annual levels, respectively.
3 Nitrate and photopigment contents, microbial diversity, and cell numbers of diatoms were analyzed in a diatom mat that had developed on the sediment surface in April 2012.

### Nitrate analysis

Three replicate sediment cores were sliced at 1-cm intervals to a total depth of 10 cm. Each slice was cut in half and each of the two half slices was transferred into its own pre-weighed 15-mL centrifugation tube. After measuring the wet weight of the samples, one vertical series of half slices was frozen at -20°C until used for photopigment analysis. The other vertical series of half slices was immediately processed for nitrate analysis. The tubes were kept on ice to minimize the microbial conversion of nitrate during the following procedure. To each sediment slice, 3 mL NaCl solution adjusted to the *in situ* salinity of 22 were added. After thorough homogenization, the sample was centrifuged at 1000 *g* for 10 min. For analysis of porewater nitrate, 0.5 mL of supernatant was collected in 2-mL centrifugation tubes and stored at -20°C. For analysis of ICNO_3_, the sample remaining in the tube was homogenized again, shock-frozen in liquid nitrogen for 5 min, and then heated to 90°C in a water bath for 10 min. This freeze-thaw sequence was repeated three times to make microbial cells in the sediment burst and thereby release ICNO_3_ [22,25]. After this procedure, the samples were centrifuged at 3000 *g* for 10 min and 0.5 mL of supernatant was collected in a 2-mL centrifugation tube and stored at -20°C. The sample remaining in the tube was dried at 65°C for 1 wk to determine the dry weight of the sediment slice.

The concentration of nitrate was measured using the VCl_3_ reduction method [31]. In the strict sense, this method detects nitrate plus nitrite; however, occasional measurements of nitrite revealed negligible concentrations. For simplicity, the nitrate plus nitrite concentrations will thus be referred to as nitrate concentrations throughout the text. The actual porewater nitrate concentration at each sediment depth was calculated from the nitrate concentration measured in the porewater extract, the water content of the sediment slice (i.e., wet weight minus dry weight), and the dilution by the added NaCl solution. The ICNO_3_ concentration at each sediment depth was calculated from the nitrate concentration measured in the cellular extract minus the calculated porewater nitrate concentration. Sedimentary nitrate concentrations are expressed per unit volume of sediment. Depth-integrated nitrate contents in the upper 10 cm of the sediment cores are expressed per unit area of sediment.

### Photopigment analysis

Sediment half slices (*see* above) were freeze-dried for 2 d. To each half slice, 5 mL ice-cold acetone was added for extraction of photopigments. Sediment and acetone were vigorously mixed and then sonicated for 5 min in a sonication bath kept at <4°C. The samples were left over night at -20°C, vigorously mixed, and centrifuged the next day at 3000 *g* for 5 min. The supernatants were filtered (Acrodisc^®^ CR 4 mm Syringe Filter with 0.45 µm Versapor^®^ Membrane, Gelman Laboratory) and filled into 2-mL glass vials. The extracted photopigments were separated by means of HPLC (Waters 2695, U.S.A.) and analyzed by a photodiode array detector (Waters 996, U.S.A.). The HPLC column (Reprosil, 350 × 4.6 mm, Dr. Maisch, Germany) was kept at 25°C, while the samples were kept at 4°C during HPLC analysis. The photopigments were separated by three different eluents (methanol: ammonium acetate (80:20), acetonitrile (90%) and ethyl acetate (100%), whose mixing ratio changed gradually during each 24-min run. In the chromatograms, chlorophyll *a* and fucoxanthin were identified according to their specific retention time and absorption spectra and the respective peaks were integrated with the Millenium®32 software (Waters, U.S.A.). Calibrations were made with serial dilutions of chlorophyll *a* and fucoxanthin stock solutions (DHI, Denmark). All procedures were made under dark conditions and using HPLC-grade chemicals. Sedimentary photopigment concentrations are expressed per unit volume of sediment. Depth-integrated photopigment contents in the upper 10 cm of the sediment cores are expressed per unit area of sediment.

### Phylogenetic analysis

Sediment from the depth interval 0-5 cm was sampled with a sterile steel core liner (inner diameter 0.7 cm, length 10 cm), transferred to a 15-mL centrifugation tube, frozen in liquid nitrogen, and stored at -80°C. Genomic DNA was extracted from 1 g of thawed and homogenized sediment using the UltraClean™ Soil DNA Isolation Kit (Mo Bio, Carlsbad, CA) supplemented with a freeze-thaw cycle to break up sturdy microbial cells. Tag-encoded FLX-amplicon pyrosequencing (TEFAP) was used to obtain partial SSU rRNA gene sequences of Bacteria, Archaea, and Eukarya. PCR, sequencing, and initial quality checking were carried out as described in [32] at the Research and Testing Laboratory (RTL, Lubbock, TX, USA). The primer sets used were 28F/519R for Bacteria, 341F/958R for Archaea, and 7F/570R for Eukarya.

Sequence reads were trimmed by removing the tags and the linker primer sequences. High-quality reads longer than 300 nt were de-replicated, sorted by length, and then clustered into operational taxonomic units (OTUs) based on >97% sequence identity using the USEARCH 6.0 software package (http://www.drive5.com/usearch, Edgar 2010). Phylotype richness was calculated using the bias-corrected estimator S_Chao1_ [33] with an on-line tool provided by the American Society of Limnology and Oceanography (http://www.aslo.org/lomethods/free/2004/0114a.html [34]).

The longest sequence of each OTU was retained for detailed sequence analysis with the ARB software package [35]. The 16S and 18S rRNA gene sequences were aligned to the non-redundant version of the SILVA SSU Ref NR 111 dataset (http://www.arb-silva.de) using the aligner integrated in ARB and manual corrections. Sequences that could not be reliably aligned were discarded from further analysis. The taxonomic affiliation of the sequences retrieved from the intertidal sediment was determined by inserting them into the SILVA SSU Ref NR 111 guide tree using maximum parsimony criteria without changing the overall tree topology. Searches against public databases were carried out using BLAST [36] to verify the taxonomic affiliation of representative sequences. All sequences were deposited in the Sequence Read Archive (SRA) under the accession numbers SAMN02222004-SAMN02222011 (www.ncbi.nlm.nih.gov/biosample).

### Diatom mat analysis

In April 2012, a dense mat of diatoms covered an extensive area of the intertidal flat near Dorum-Neufeld. The mat was carefully sampled with a sterile spatula, transferred into a sterile centrifuge tube, and transported on ice to the laboratory. The mat sample was homogenized with a sterile spatula and subdivided into seven equally sized parts, three for nitrate and photopigment analysis, three for diatom cell counting, and one for pyrosequencing. Nitrate and photopigment analysis and pyrosequencing were made as described for the sediment samples. For diatom cell counting, 5 cm^3^ of each mat subsample was transferred into a sterile glass flask and vigorously mixed in 250 mL sterile NaCl solution (salinity of 22) for 1 h. The diatom cells remained intact during this procedure. In subsamples of the slurries, the diatom cell number was determined using a Fuchs-Rosenthal counting chamber. Nitrate and photopigment contents and diatom abundance are expressed per unit volume of mat. Assuming that ICNO_3_ in the mat was exclusively stored by diatoms, the average cell-volume-specific ICNO_3_ concentration (expressed in mmol per L of cell volume) was calculated as follows: The ICNO_3_ concentration in the mat was divided by the diatom cell number in the mat and then related to the biovolume of small (0.1 pL) and large (0.5 pL) diatom cells typically occurring in coastal marine sediments.

## Results

### Seasonal nitrate distribution

**Figure 1:**
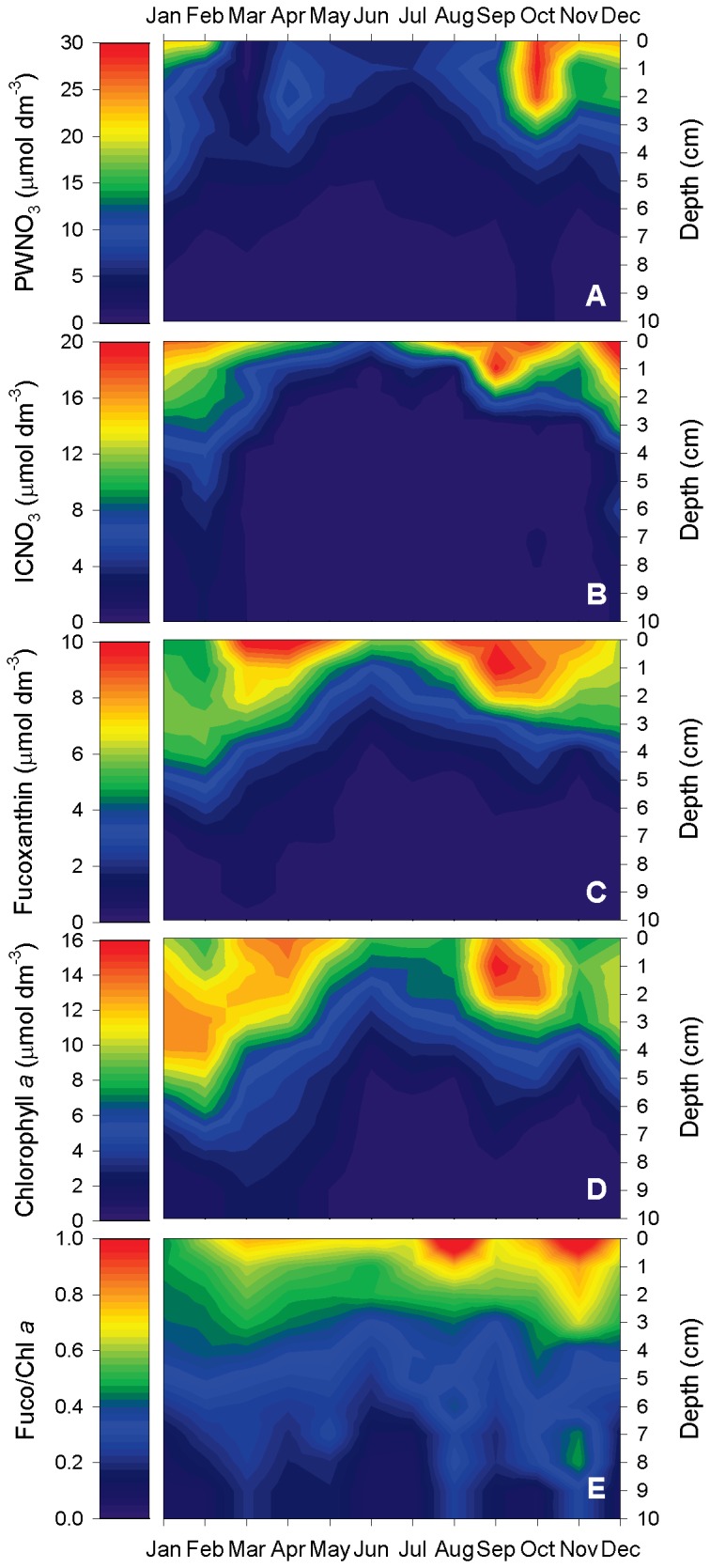
Seasonality of nitrate and photopigment concentrations in intertidal sediment. A) Porewater nitrate (PWNO_3_), B) intracellular nitrate (ICNO_3_), C) fucoxanthin, D) chlorophyll *a*, and E) the molar fucoxanthin-to-chlorophyll *a* ratio (Fuco/Chl *a*) were determined in monthly intervals in an intertidal flat of the German Wadden Sea. Data were collected in 1-cm depth intervals. For each month, the means of three replicate sediment cores are shown.

Concentration levels and penetration depths in sediments of extra- and intracellular nitrate showed similar trends and were both generally higher during the cold season than during summer (Figure 1A+B, Figure S1). Porewater nitrate concentrations were high at the sediment surface (up to 29.6 µmol NO_3_^-^ dm^-3^ sediment) and decreased with depth (Figure 1A). Occasionally, subsurface concentration maxima were observed (e.g., in March and April, Figure S1). Below 7 cm depth, porewater concentrations were always negligible (Figure S1). The seasonal and depth distribution of ICNO_3_ was similar to that of porewater nitrate: ICNO_3_ concentrations of up to 22.3 µmol dm^-3^ were measured at the sediment surface and negligible values were mostly reached below 5 cm sediment depth (Figure 1B). A small subsurface concentration maximum was only observed in September (Figure S1).

**Figure 2:**
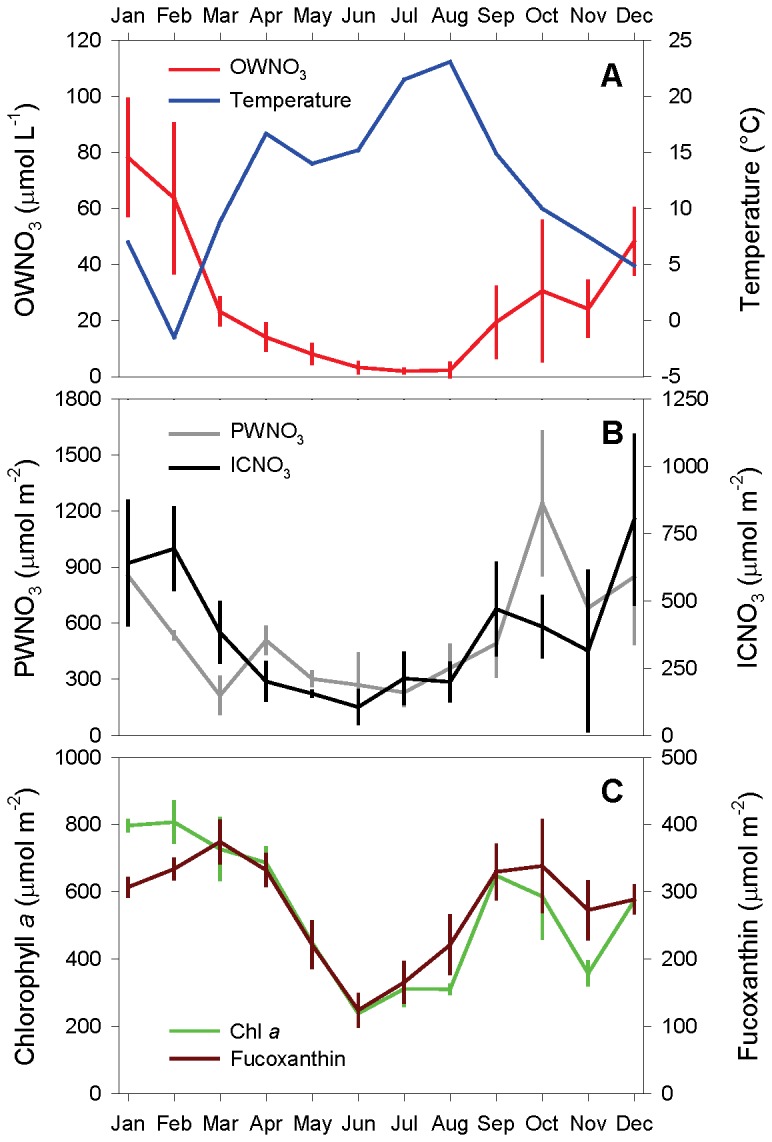
Seasonality of the different nitrate pools and photopigment contents in intertidal sediment. A) Overlying-water nitrate (OWNO_3_) and temperature, B) depth-integrated contents of porewater nitrate (PWNO_3_) and intracellular nitrate (ICNO_3_), and C) depth-integrated contents of fucoxanthin and chlorophyll *a* were determined in monthly intervals for the upper 10 cm of an intertidal flat of the German Wadden Sea. For each month, the means ± s.d. of three replicate sediment cores or 3-5 water samples are shown.

Nitrate concentration in the water overlying the sediment at low tide (OWNO_3_) ranged from 0.6 to 78 µmol L^-1^. Temperature and OWNO_3_ showed opposing seasonal trends (Figure 2A). In contrast, the depth-integrated contents of both porewater and intracellular nitrate showed the same seasonal trends as OWNO_3_ (Figure 2A+B). Accordingly, the depth-integrated contents of ICNO_3_ were significantly correlated to overlying water concentrations and depth-integrated porewater contents of nitrate (both positive) and to sediment temperature (negative) (Table 1). Depth-integrated contents of porewater and intracellular nitrate were similarly high throughout the year, indicating that the total nitrate pool in the sediment was estimated ca. two times higher when ICNO_3_ contents were included (Figure 2B).

**Table 1:** Correlation between the depth-integrated contents of intracellular nitrate and other variables in an intertidal flat of the German Wadden Sea.

**Variable**	**Temperature**	**OWNO_3_**	**PWNO_3_**	**Chlorophyll *a***	**Fucoxanthin**
**ICNO_3_**	-0.688**	+0.737**	+0.420*	+0.596**	+0.523**
**Fucoxanthin**	-0.422**	+0.593**	+0.362*	+0.851**	
**Chlorophyll *a***	-0.582**	+0.731**	+0.396*		
**PWNO_3_**	-0.515**	+0.704**			
**OWNO_3_**	-0.921**				

Spearman’s coefficients for non-linear correlations are shown. Asterisks indicate significant correlations at the 0.05 (*) and 0.01 (**) level.

N = 36 sediment cores collected in monthly intervals (i.e., 12 months × 3 cores)

ICNO_3_: Intracellular nitrate, OWNO_3_: Overlying-water nitrate, PWNO_3_: Porewater nitrate

### Seasonal photopigment distribution

Fucoxanthin concentrations were high at the sediment surface (up to 10.3 µmol dm^-3^) and decreased with depth (Figure 1C). Concentration levels and penetration depths were especially high during spring and fall, intermediate in winter, and lowest during summer (Figure 1C, Figure S2). Subsurface concentration maxima were common, but not very pronounced (Figure S2). Below 7 cm depth, fucoxanthin concentrations were always negligible (Figure S2).

Chlorophyll *a* concentrations were high at the sediment surface (up to 13.9 µmol dm^-3^), but often even higher in subsurface layers (up to 16.0 µmol dm^-3^) (Figure 1D, Figure S2). Only below 4 cm depth, chlorophyll *a* concentrations always decreased to lower values (Figure 1D, Figure S2). Spring and fall maxima in chlorophyll *a* concentration were clearly discernible, but high concentration levels and penetration depths were also observed in January (Figure 1D). Chlorophyll *a* concentrations were sometimes even substantial at 10 cm depth (Figure S2). The molar fucoxanthin-to-chlorophyll *a* ratio (Fuco/Chl *a*) generally decreased with depth in an often linear fashion (Figure 1E, Figure S3). At the sediment surface, Fuco/Chl *a* ranged from 0.45 to 1.20, whereas at 10 cm depth, it ranged from 0.08 to 0.29 (Figure S3). Seasonal changes of Fuco/Chl *a* were negligible (Figure 1E).

The depth-integrated contents of both fucoxanthin and chlorophyll *a* exhibited maxima in spring and fall and a pronounced minimum in summer (Figure 2C). Thus, pigment contents and nitrate in the overlying water and the sediment showed similar seasonal trends (Figure 2A–C). Accordingly, the depth-integrated contents of fucoxanthin and chlorophyll *a* were significantly correlated to all nitrate variables (positive) and to sediment temperature (negative) (Table 1). Depth-integrated contents of fucoxanthin and chlorophyll *a* were closely correlated to each other (Table 1).

### Intracellular nitrate relative to fucoxanthin

**Figure 3:**
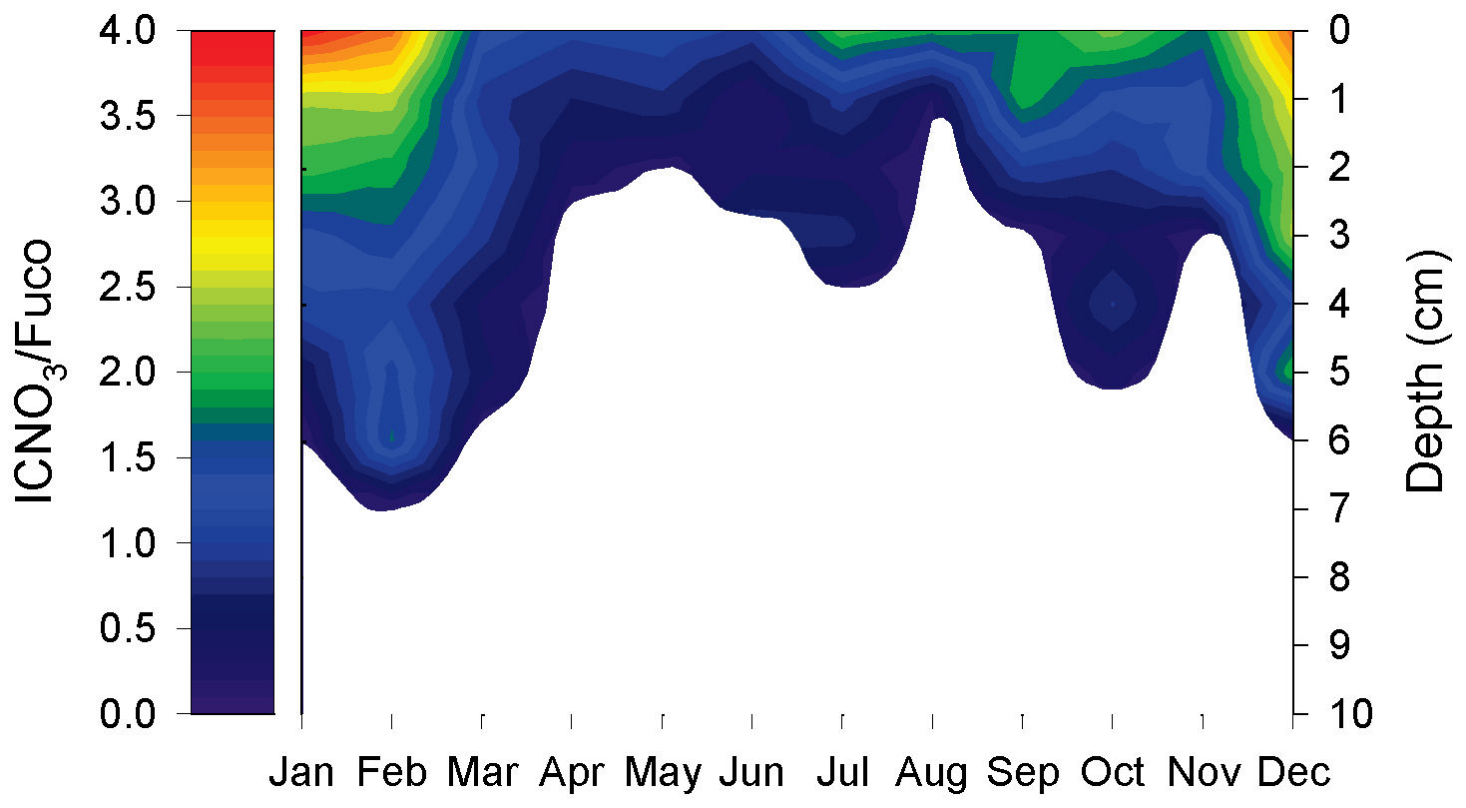
Seasonality of the molar intracellular-nitrate-to-fucoxanthin ratio (ICNO_3_/Fuco) in intertidal sediment. ICNO_3_/Fuco was calculated for fucoxanthin concentrations >1.5 µmol dm^-3^; the white area of the plot is therefore undefined. The primary data were collected in 1-cm depth intervals. For each month, the means of three replicate sediment cores are shown.

Assuming that the occurrence of sedimentary ICNO_3_ is exclusively due to nitrate storage by diatoms and that fucoxanthin is a truly quantitative biomarker for diatoms (*see* below), the molar intracellular-nitrate-to-fucoxanthin ratio (ICNO_3_/Fuco) is a measure for diatom-specific ICNO_3_. However, this ratio is not useful for particularly low fucoxanthin concentrations because then ICNO_3_/Fuco increases to unrealistically high values. Here, ICNO_3_/Fuco was calculated only for fucoxanthin concentrations >1.5 µmol dm^-3^. At all times of the year, ICNO_3_/Fuco was high at the sediment surface (up to 3.9) and decreased with depth (Figure 3, Figure S4). Maximum ICNO_3_/Fuco values were observed during winter, while intermediate and low ICNO_3_/Fuco values prevailed during summer and spring, respectively (Figure 3, Figure S4). The annual average (± s.d.) of ICNO_3_/Fuco in the top 1 cm of the sediment was 2.1 (± 1.0).

### Nitrate and photopigments in the diatom mat

ICNO_3_ concentrations in the diatom mat sampled in April 2012 were on average 65.1 µmol dm^-3^ and thus by far higher than the maximum ICNO_3_ concentration measured in the sediment (Table 2). The same was true for fucoxanthin, but not for chlorophyll *a* (Table 2). The average diatom abundance in the mat was 1.40 × 10^10^ cells dm^-3^ (Table 2). Assuming that ICNO_3_ in the mat was exclusively stored by diatoms, the average cell-volume-specific ICNO_3_ concentration in diatoms might have ranged between 9.3 and 46.7 mmol L^-1^, if all diatom cells had a biovolume of 0.5 and 0.1 pL, respectively (Table 2). Fuco/Chl *a* in the diatom mat was 1.64 and thus higher than the maximum ratio of 1.20 observed in the sediment. ICNO_3_/Fuco in the diatom mat was 2.4 and thus lower than the maximum ratio of 3.9 observed in the sediment, but close to the annual average of ICNO_3_/Fuco in the top 1 cm of the sediment.

**Table 2:** Nitrate and photopigment concentrations, diatom abundance, and cell-specific nitrate concentrations in a diatom mat covering an intertidal flat of the German Wadden Sea.

**Concentration or abundance in diatom mat**				**Concentration in diatoms**	
**ICNO_3_**	**Fucoxanthin**	**Chlorophyll *a***	**Diatoms**	**ICNO_3_**	
**(µmol dm^-3^)**	**(µmol dm^-3^)**	**(µmol dm^-3^)**	**(cells dm^-3^)**	**(mmol L^-1^)**	
				**Cell volume**	**Cell volume**
				**0.1 pL**	**0.5 pL**
65.1	27.8	16.8	1.40 × 10^10^	46.7	9.3
(9.8)	(8.0)	(4.0)	(1.38 × 10^9^)	(6.1)	(1.2)

Cell-volume-specific ICNO_3_ concentrations in diatoms were calculated for cell volumes of 0.1 and 0.5 pL, typical of *Cylindrotheca* sp. and *Amphora* sp., respectively, which were abundant in the intertidal flat. Mean values (s.d.) of 3 replicate mat samples are given.

### Prokaryotic and eukaryotic diversity

In the sediment samples, the communities of the three domains of life were exhaustively covered by the pyrosequencing approach as indicated by OTU numbers very close to the richness estimate (Table 3, Table S1). The lower OTU numbers in intertidal sediment collected in December are probably due to the lower sequencing depth for this sample (Table 3).

**Table 3:** Number of 16S and 18S rDNA pyroreads and operational taxonomic units (OTUs) in intertidal sediment and diatom mat samples collected in the German Wadden Sea.

**Sample**	**Sediment (Dec 2011)**				**Sediment (Jun 2011)**				**Diatom mat (Apr 2012)**			
**Domain**	**Archaea**	**Bacteria**	**Eukarya**		**Archaea**	**Bacteria**	**Eukarya**		**Archaea**	**Bacteria**	**Eukarya**	
**Total reads**	2487	2633	3645		14457	2929	7584		NA	3836	6650	
Cleaned reads*	618	671	424		2266	868	1056		NA	3007	1096	
**Total OTUs**	254	346	152		705	397	410		NA	338	157	
Cleaned OTUs**	13	314	137		27	392	357		NA	326	153	
			**PF**	**MZ**			**PF**	**MZ**			**PF**	**MZ**
			75	62			275	82			107	46

^*^ Cleaning of reads included trimming (i.e., removal of tags and linker primer sequences), de-replication, and discarding of sequences shorter than 300 nt using USEARCH 6.0 [62].

^**^ Cleaning of OTUs included discarding OTU sequences that could not be aligned to the target sequence and OTUs that affiliated with Bacteria, despite primers for Archaea were used.

PF: Protista + Fungi, MZ: Metazoa, NA: Not analyzed

**Figure 4:**
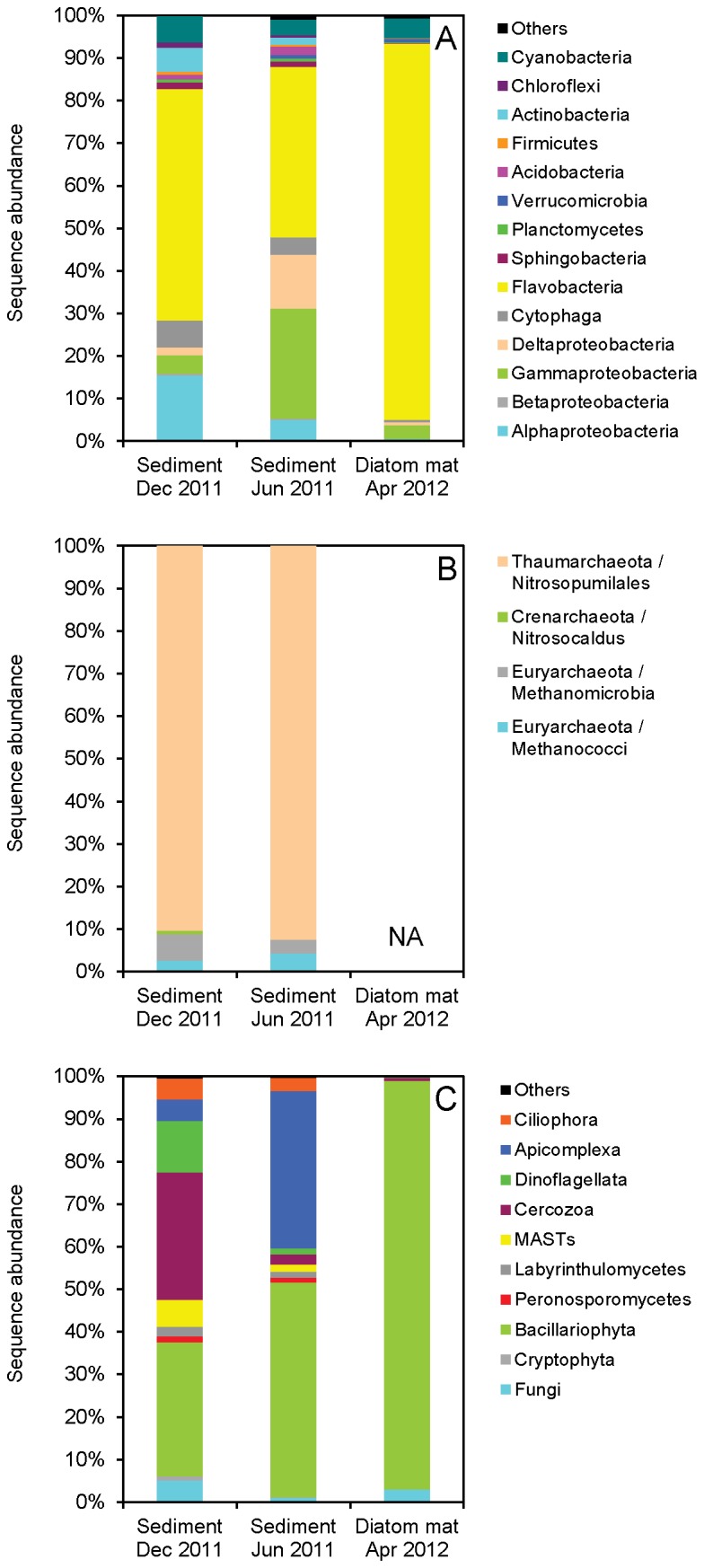
Diversity of Bacteria, Archaea, and Eukarya in intertidal sediment. Taxonomic affiliation and relative sequence abundance of the most common A) bacterial, B) archaeal, and C) eukaryotic (here: Protista and Fungi) groups were retrieved by pyrosequencing of SSU rRNA genes in DNA extracted from intertidal sediment sampled in December 2011 and June 2011, and from an intertidal diatom mat sampled in April 2012. NA: Not analyzed.

Within Bacteria and Archaea, the highest relative sequence abundances were found for Flavobacteria, α-, γ-, δ-Proteobacteria, and Thaumarchaeota (Figure 4A+B). Notably, no sequences affiliated with Thiotrichaceae (within γ-Proteobacteria), which comprise many nitrate-storing sulfur bacteria, were retrieved by pyrosequencing. In agreement with this finding, *Beggiatoa* , *Thioploca* , and *Thiomargarita* were not detected by microscopy.

Within Eukarya (excl. Metazoa), Bacillariophyta (which comprise all diatoms) were the dominant phylum in all three samples in terms of relative sequence abundance (Figure 4C). In sediment collected in December and June, Cercozoa and Apicomplexa sequences, respectively, were similarly abundant as Bacillariophyta sequences. In the diatom mat, the quantitative dominance of Bacillariophyta sequences was expectedly overwhelming and aside from Bacillariophyta sequences only Fungi sequences had a significant share (3%) of the total sequence abundance. The most abundant Bacillariophyta sequences in the diatom mat affiliated with the genera *Gyrosigma* (ca. 50% of the total number of Bacillariophyta sequences), *Nitzschia* (ca. 12%), and *Navicula* (ca. 5%), which agreed well with qualitative microscopic observations.

**Figure 5:**
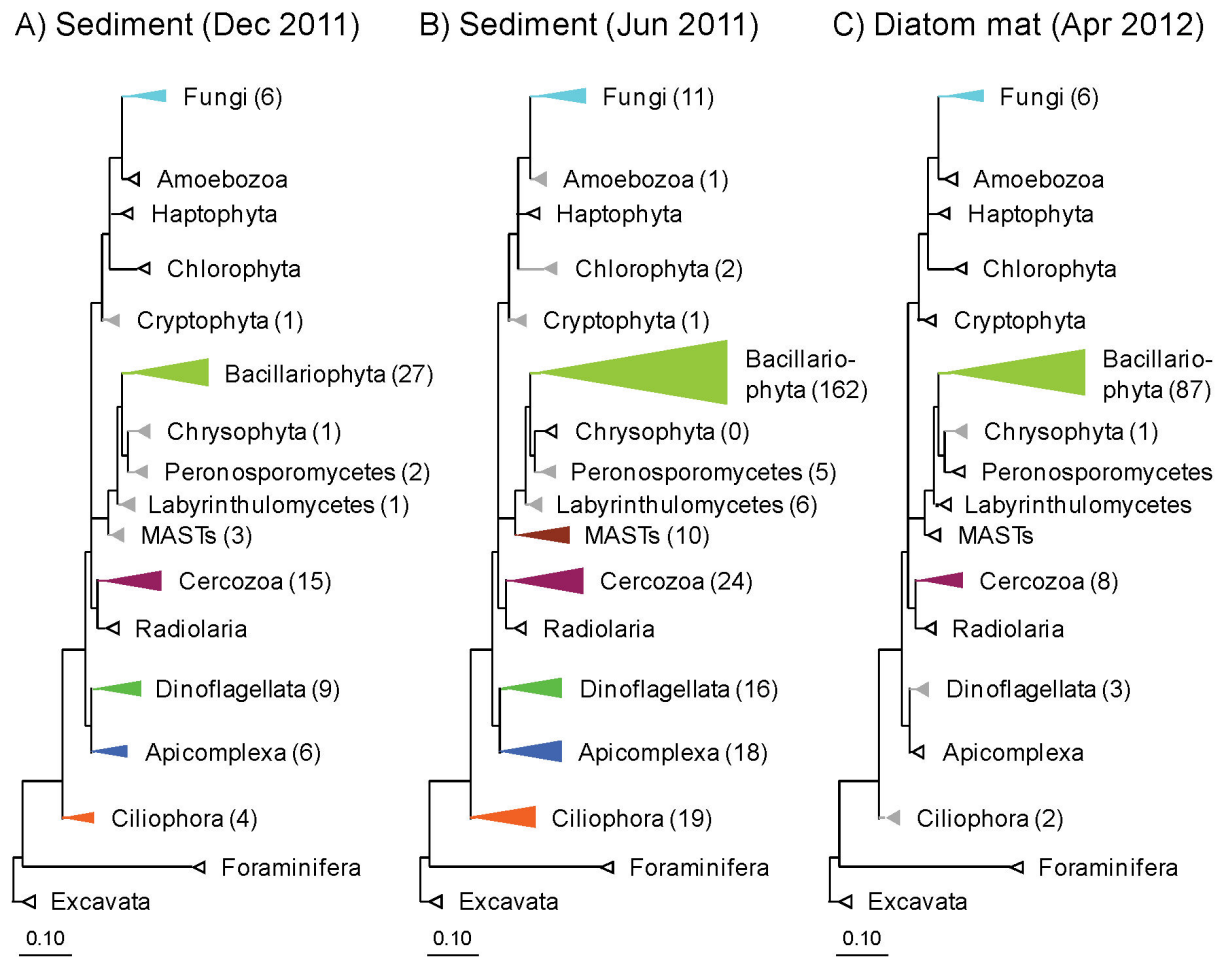
Eukaryotic diversity in intertidal sediment. 18S rDNA-based phylogenetic reconstruction of eukaryotic diversity (here: Protista and Fungi) in intertidal sediment sampled in A) December 2011 and B) June 2011, and C) in an intertidal diatom mat sampled in April 2012. Phyla with more than three OTUs are shown as colored leaves, phyla with 1-3 OTUs as grey leaves, and unrepresented phyla as white leaves. The size of the colored leaves is proportional to the number of OTUs in each phylum which is given in parentheses. The scale bar represents the number of substitutions for a unit branch length.

Bacillariophyta dominated in all three samples also in terms of OTU richness (Figure 5). Other phyla with high OTU richness were Cercozoa, Fungi, Dinoflagellata, Apicomplexa, and Ciliophora (Figure 5). Within Bacillariophyta, all three classes (i.e., Cosinodiscophyceae, Fragilariophyceae, and Bacillariophyceae) were represented in both the sediment and the diatom mat (Figure S5). Sequences affiliating with diatom species known to store nitrate intracellularly (e.g., *Amphora* *coffeaeformis* , *Thalassiosira* *weissflogii* *,* *Nitzschia* *punctata* ) were found in the sediment and/or the diatom mat (Figure S5).

Strikingly, nitrate-storing Foraminifera were not detected by pyrosequencing in any of the samples (Figure 4C and Figure 5), despite the fact that at least a few tests of Foraminifera were detected by microscopy in the sediment, but not in the diatom mat. Eukaryotic phyla not included in Figure 4C and Figure 5, but retrieved by pyrosequencing comprised the metazoan phyla Nematoda, Crustacea, Tardigrada, and Plathelminthes. Sequences of Nematoda, mainly affiliating with the families Oncholaimidae and Xyalidae, were highly abundant in the sediment (25-80% of total Eukarya sequences), but not in the diatom mat (11.5%).

## Discussion

### Seasonal nitrate distribution

The intertidal flat near Dorum-Neufeld was characterized by considerable spatial-temporal variation of three different nitrate pools: 1) nitrate freely dissolved in the water column, 2) nitrate freely dissolved in the sediment porewater, and 3) nitrate contained within living microbial cells. These nitrate pools are interconnected as can be concluded from the similar vertical distribution of porewater and intracellular nitrate in the sediment and from the similar time course of water column, porewater, and intracellular nitrate over an annual cycle. The highest porewater and intracellular nitrate concentrations were found close to the sediment surface, which suggests that nitrate availability in the water column is a major control of the size of the nitrate pools in the sediment. Additionally, high nitrate concentrations in the upper sediment layers may be caused by nitrification activity at the oxic sediment surface. Microsensor measurements in laboratory-incubated sediment taken from the same intertidal flat indeed revealed a tight correlation between nitrification activity and ICNO_3_ concentrations [25]. However, in the field-collected sediment cores studied here, porewater and intracellular nitrate concentrations were substantial at sediment depths greatly exceeding the oxygen penetration depth of <0.5 cm in the retrieved cores (data not shown). Advective transport of nitrate-rich (and oxygenated) water into the sediment is a more likely explanation than nitrification for the deep occurrence of nitrate in sandy intertidal sediment [37]. In the sandy-to-silty sediment studied here, however, advective porewater transport is probably of minor importance, especially during low tide when significant water flow above the sediment surface is absent. Nevertheless, entrainment of nitrate-rich water into deeper layers may well occur due to mixing forces such as bioturbation, bioirrigation, deposition-resuspension events, and ripple movement [38–41]. The deep occurrence of ICNO_3_ may generally also result from vertical migration of nitrate-storing microorganisms such as *Thioploca* [6], *Beggiatoa* [42], and Foraminifera [43]. In the following, however, we argue that the ICNO_3_ pool in the intertidal sediment studied here and possibly in other microphytobenthos-dominated coastal marine sediments is mainly due to diatoms either actively migrating or passively being buried in the sediment.

ICNO_3_ concentrations in the intertidal sediment of the Wadden Sea were similar to those found in other coastal marine sediments [22,24], but lower than the 60-175 µmol dm^-3^ encountered in sediments densely colonized by large sulfur bacteria [10,23,44,45] or Foraminifera [12]. In some of these studies, the possible nitrate storage by benthic microalgae or settled phytoplankton was discussed, but not correlated to a diatom-specific biomarker like the photopigment fucoxanthin. If the phototrophic diatoms or other microalgae are significantly involved in benthic ICNO_3_ storage, then a certain seasonality of the ICNO_3_ pool can be expected. In fact, Lomstein et al. [22] found high benthic ICNO_3_ concentrations directly after the settlement of a phytoplankton bloom in spring and low concentrations during fall and winter. In contrast, Garcia-Robledo et al. [24] found higher ICNO_3_ concentrations in January than in July in intertidal sediment of the Mediterranean Sea. The latter observation agrees well with that made in the intertidal sediment of the Wadden Sea, with high and low ICNO_3_ concentrations measured throughout the cold and warm season, respectively. A plausible explanation for high benthic ICNO_3_ concentrations during the cold season is the high concentration of nitrate in the water column. In temperate zones, high winter nitrate concentrations in the water column of lakes and streams are due to high rates of nitrate leaching from arable land and low rates of nitrate consumption by primary producers and denitrifiers in aquatic ecosystems [46]. Likewise, intertidal sediments receive high nitrate loads through riverine inputs into the coastal zone especially during the cold season [47,48].

### Seasonal photopigment distribution

The microphytobenthos of the intertidal sediment was generally diatom-dominated as indicated by molar Fuco/Chl *a* ratios >0.5 in the upper 3 cm of the sediment, microscopic observations, and the pyrosequencing results. In a number of other studies on intertidal sediments, molar Fuco/Chl *a* ratios of 0.50-0.95 were taken as an indication for diatom dominance [26–29]. In aphotic sediments of greater water depth, the molar Fuco/Chl *a* ratio can be significantly higher (e.g., 1.48 in coastal waters [49]) because of massive sedimentation and burial of diatom-dominated phytoplankton. We found an even higher value of 1.64 in the diatom mat established on the surface of the intertidal sediment, where macro- and microscopic evidence of diatom dominance was most striking. The molar Fuco/Chl *a* ratio decreased with sediment depth, which may be due to a stronger down-regulation or degradation of fucoxanthin compared to chlorophyll *a* under dark, anoxic conditions as was observed in axenic diatom strains (unpublished data AK). In five benthic and pelagic diatom strains, the molar Fuco/Chl *a* ratio decreased on average (± s.d.) from 0.72 ± 0.11 to 0.42 ± 0.08 within one week of dark, anoxic incubation during which all strains remained viable. Alternative explanations would be physiological shifts in Fuco/Chl *a* in response to low-light conditions or the depth-specific occurrence of diatom populations that differ in Fuco/Chl *a*.

Both chlorophyll *a* and fucoxanthin were invariably present in high concentrations in permanently dark sediment layers. This common phenomenon is explained by active vertical migration of diatoms combined with their dark survival potential [20,49–51] or by passive burial via bioturbation, deposition-resuspension events, and ripple movement [38–40]. A possible consequence of the occurrence of viable diatoms in dark sediment layers is the entrainment of nitrate into these layers along with the diatom cells. The very good spatial-temporal overlap of diatom pigments and ICNO_3_ in the intertidal sediment of the Wadden Sea strongly supports the idea that diatoms constitute important reservoirs of nitrate throughout the upper few centimeters of intertidal sediment. In muddy intertidal sediment of the Mediterranean Sea, a similar spatial correlation of chlorophyll *a* (fucoxanthin was not measured) and ICNO_3_ was found, albeit with much steeper vertical gradients than in the sandy-to-silty sediment of the Wadden Sea [24].

The spatial correlation of diatom pigments and ICNO_3_ was complemented by a temporal correlation revealed during a complete annual cycle. Just like the ICNO_3_ concentrations, both chlorophyll *a* and fucoxanthin concentrations showed minima and maxima during the warm and cold season, respectively. While this pattern is at first counterintuitive, it can be explained by low nutrient concentrations and high grazing pressure in the summer and the opposite situation in the winter [26,27,52]. Additionally, it can be speculated that high water-column and porewater nitrate concentrations in the winter allow diatoms to accumulate nitrate intracellularly and to survive in deep, anoxic layers that provide shelter from freezing [20]. In some intertidal flats, the decrease in diatom abundance during the warm season is accompanied by an increase in the abundance of cyanobacteria, possibly because they are not affected by silicate limitation like diatoms that need silicate for the formation of their frustule and because diazotrophic cyanobacteria fix nitrogen [27,53]. The pyrosequencing data, however, did not indicate that cyanobacteria gained in importance in the Wadden Sea sediment during the warm season. Instead, the pigment data revealed two smaller diatom maxima, one in spring and one in fall. These diatom maxima may go back to spring and fall phytoplankton blooms in the North Sea followed by sedimentation of diatoms onto intertidal flats [27,30]. Interestingly, only the pigment maximum observed in fall was accompanied by a maximum in benthic ICNO_3_ concentrations, which could mean that ICNO_3_ concentrations of pelagic diatoms were high during fall and low during spring. Finally, it needs to be noted that the seasonal variation in benthic fucoxanthin concentrations may in part result from the change of intracellular fucoxanthin concentrations of the diatoms in adaptation to high- or low-light conditions [54].

### Diatoms as cellular nitrate reservoirs

**Figure 6:**
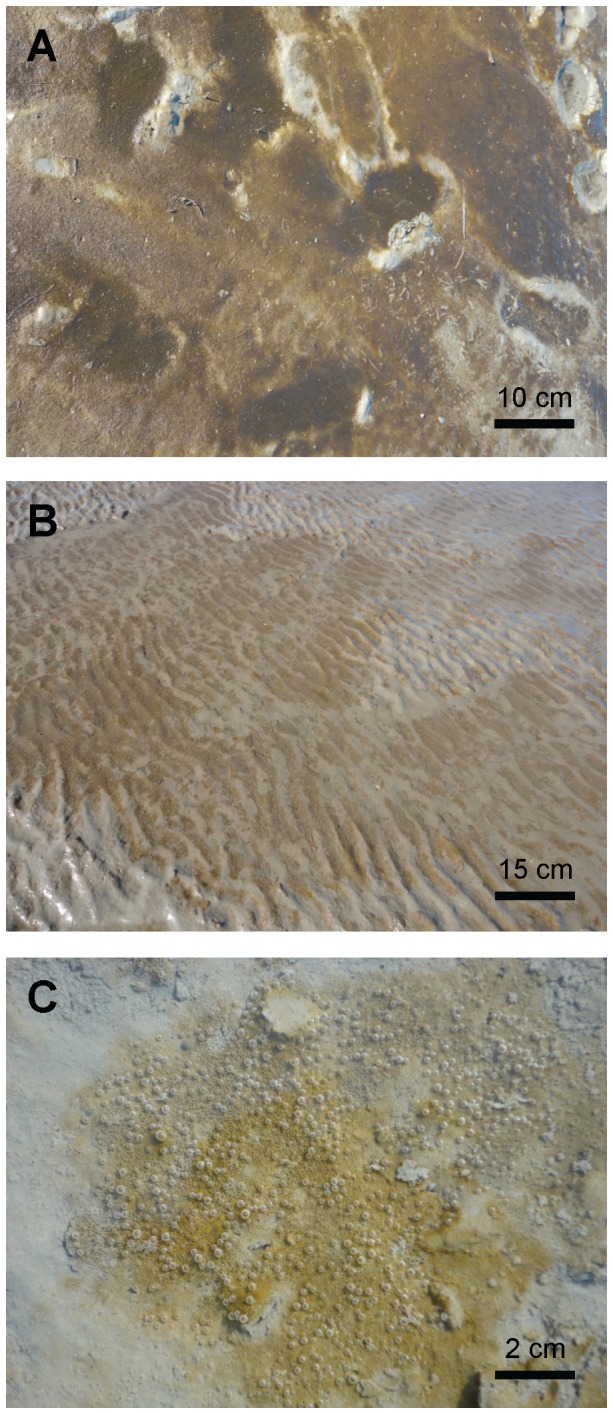
Diatom mat covering intertidal sediment. In April 2012, a diatom mat established on the sediment surface of an intertidal flat in the German Wadden Sea near Dorum-Neufeld (53°45'N, 8°21'E). A) B), and C) show images taken at different distances from the sediment surface (*see* scale bars). Brown patches correspond to a dense lawn of diatom cells. Gas bubbles are due to oxygen oversaturation close to the surface of the photosynthetically active diatom mat.

The tight spatial-temporal correlation between benthic concentrations of fucoxanthin and ICNO_3_ strongly suggests that diatoms are important cellular nitrate reservoirs in intertidal sediments. The eye-catching diatom mat that had developed on the sediment surface in April 2012 clearly enforced this view because fucoxanthin and ICNO_3_ concentrations in the mat exceeded those in bare sediment in about equal measures. Even more importantly, the mat was visibly dominated by diatoms (Figure 6), which was also corroborated by the pyrosequencing results. Diatoms were represented by overwhelming numbers of both OTUs and sequence reads compared to other Protista and Fungi. The diatom community in the mat, but also in the sediment, comprised three genera for which ICNO_3_ storage was shown in axenic strains (i.e., *Amphora* , *Thalassiosira* , and *Nitzschia* [20]). Importantly, sequences affiliating with the nitrate-respiring *Amphora* *coffeaeformis* were abundant too [20]. At the same time, the absence of other known nitrate-storing microorganisms (i.e., Foraminifera, Gromiida, and Thiotrichaceae) from the diatom mat was confirmed by both pyrosequencing and microscopy. In the bare sediment, however, the absence of other known nitrate-storing microorganisms was only confirmed by pyrosequencing, while a few tests of Foraminifera were detected by microscopy. Neither the taxonomic affiliation, nor the viability of these Foraminifera was further analyzed. Bacterial and Archaeal sequence reads were dominated by Flavobacteria and Nitrosopumilales, respectively. Flavobacteria have previously been revealed as the most abundant Bacterial phylum in Wadden Sea sediment, which was attributed to their metabolic ability to degrade polymeric organic substances [55,56]. The coexistence of diatoms and Flavobacteria, especially in the diatom mat, may thus be founded on the release of polymeric organic substances by the diatoms. Nitrosopumilales were represented by the ammonia-oxidizing *Candidatus* Nitrosopumilus that is highly abundant in marine surface waters, but was originally isolated from marine sediments [57]. Flavobacteria and Nitrosopumilales are not known to store nitrate intracellularly, which is also not very likely because of their small cell size.

Taken together, the community analysis singles out diatoms as the most likely taxonomic group that is responsible for the observed ICNO_3_ pool in intertidal sediment. Additionally, the community analysis gives hints to possibly nitrate-storing diatoms such as the large-celled *Gyrosigma* sp. that should now be investigated in pure culture. Its occurrence in diatom mats growing on top of intertidal sediment is apparently common [28].

Based on cell counts in the diatom mat and a number of assumptions, the range of cell-specific ICNO_3_ concentrations (i.e., 9.3-46.7 mmol L^-1^) indicates that the diatoms of the Wadden Sea sediment accumulate nitrate to concentrations that are 2-3 orders of magnitude higher than ambient concentrations. This range of cell-specific ICNO_3_ concentrations is at the lower end of that found in isolated or enriched benthic and pelagic diatoms (*see* references above). Foraminifera, Gromiida, and Thiotrichaceae, however, commonly store nitrate in concentrations of several hundred millimolar (*see* references above). Nevertheless, the total contribution of the ubiquitous diatoms to benthic ICNO_3_ pools may exceed that of other nitrate-storing microorganisms that are only abundant in specific and spatially confined benthic habitats.

### Fate of diatom-stored nitrate

If one accepts that diatoms have ICNO_3_ stores not only in the upper water column [17–19] and at the sun-lit sediment surface [24], but also in aphotic sediment layers, then the question arises what the ultimate fate of this nitrate may be. Nitrate is assimilated by diatoms that are photosynthetically active [17–19], which is the case in euphotic water and sediment layers. In dark and anoxic sediment layers, the accumulation of ICNO_3_ may serve to produce energy via anaerobic nitrate respiration. Diatoms have recently been shown to anaerobically reduce ICNO_3_ to ammonium which is simultaneously released from the cell [20]. This finding extended the rather short list of Eukarya capable of anaerobic nitrate respiration by a group of organisms that occur ubiquitously and often at high abundance in aquatic ecosystems. Both pelagic and benthic diatoms may use their ICNO_3_ stores for dissimilatory energy production when exposed to dark and anoxic conditions deeper in the water column or in the sediment, but direct evidence for the occurrence of this process under *in situ* conditions is still lacking. It could be argued that the diatom community of the Wadden Sea sediment is not directly involved in anaerobic nitrate reduction because ICNO_3_ concentrations were still high in relatively deep sediment layers. In contrast, the diatom strains tested under laboratory conditions use up their nitrate stores within less than one day when exposed to dark and anoxic conditions. However, in contrast to the controlled laboratory incubations, intertidal sediments are characterized by strong dynamics with respect to light, oxygen, and nitrate availability due to porewater advection, deposition-resuspension events, bioturbation, and photosynthesis [41]. Diatoms may thus experience recurring opportunities to refill their ICNO_3_ stores, most likely aided by their ability to migrate vertically in the sediment [51]. In the Wadden Sea sediment, the molar ICNO_3_/Fuco ratio decreased with depth, which at first sight suggests that diatoms take up nitrate close to the sediment surface and consume nitrate in deeper, anoxic sediment layers. However, also PWNO_3_ concentrations decreased with depth, which suggests that ICNO_3_ is in equilibrium with PWNO_3_, but is enriched relative to PWNO_3_ by 2-3 orders of magnitude. The decrease of ICNO_3_/Fuco with sediment depth can thus not be used as evidence for the direct involvement of diatoms in anaerobic nitrate reduction.

So far, one benthic and one pelagic diatom strain are known to anaerobically reduce ICNO_3_ to ammonium ( [20], unpublished data AK). If this metabolic pathway is widespread, then diatoms would not remove fixed nitrogen from the intertidal ecosystem, but only convert one form of fixed nitrogen into another one. In this respect, diatoms would resemble those Thiotrichaceae that couple sulfide oxidation to nitrate reduction to ammonium, for instance, *Beggiatoa* [10] or *Thioploca* [11]. By contrast, bacteria capable of denitrification or anaerobic ammonium oxidation (anammox) remove fixed nitrogen from the ecosystem, and the same holds true for Foraminifera that produce dinitrogen or nitrous oxide from nitrate [12–15].

So far, neither evidence, nor measurements of anaerobic nitrate reduction by diatoms in intertidal sediments exist. Thus, the potential contribution of diatoms to anaerobic nitrate reduction in the Wadden Sea sediment can only be estimated based on the span of cell-specific rates of ammonium production by *A* *. coffeaeformis* [20] and the depth-integrated fucoxanthin contents as well as the fucoxanthin-to-cell conversion factor obtained in this study. During the summer and the winter, diatoms may produce 9-183 and 28-553 µmol NH_4_^+^ m^-2^ h^-1^, respectively. These rates are in the same range as areal denitrification rates reported for Wadden Sea and other intertidal sediments (8-48 [47], 1-55 [58], 10-280 [59], and 230-470 µmol N_2_–N m^-2^ h^-1^ [60]). Very recently, the areal denitrification rate in the intertidal flat near Dorum-Neufeld was measured to be 88 µmol N_2_–N m^-2^ h^-1^ [61], which is also within the estimated range of areal nitrate ammonification rates of diatoms. Additionally, diatoms with intact ICNO_3_ stores, possibly filled in the water column, may also decay and be mineralized in the sediment. Nitrate released from lysing diatom cells is available to the benthic microbial community, which may increase the rate of all pathways of anaerobic nitrate or nitrite reduction such as denitrification, anammox, and dissimilatory nitrate reduction to ammonium [22]. Thus, nitrate-storing diatoms may have a so far overseen impact on the coastal marine nitrogen cycle that needs to be further investigated directly in the environment.

## Supporting Information

Table S1 **Number of operational taxonomic units (OTUs) observed and estimated in intertidal sediment and diatom mat samples from the German Wadden Sea.**
(DOC)

Figure S1 **Seasonality of porewater nitrate (PWNO_3_) and intracellular nitrate (ICNO_3_) concentrations determined in monthly intervals in an intertidal flat of the German Wadden Sea.** For each month, means ± s.d. of 3 replicate sediment cores are shown.
(TIFF)

Figure S2 **Seasonality of chlorophyll *a* (Chl *a*) and fucoxanthin (Fuco) concentrations determined in monthly intervals in an intertidal flat of the German Wadden Sea.** For each month, means ± s.d. of 3 replicate sediment cores are shown.
(TIFF)

Figure S3 **Seasonality of the molar fucoxanthin-to-chlorophyll *a* ratio (Fuco/Chl *a*) in intertidal sediment sampled in monthly intervals in the German Wadden Sea.** For each month, means ± s.d. of 3 replicate sediment cores are shown.
(TIFF)

Figure S4 **Seasonality of the molar intracellular-nitrate-to-fucoxanthin (ICNO_3_/Fuco) ratio in intertidal sediment sampled in monthly intervals in the German Wadden Sea.** For each month, means ± s.d. of 3 replicate sediment cores are shown.
(TIFF)

Figure S5 **18S rDNA-based phylogenetic reconstruction of Bacillariophyta in intertidal sediment and an intertidal diatom mat.** The number of OTUs in each sequence cluster is given in the blue (Sediment, December 2011), red (Sediment, June 2011), and green boxes (Diatom mat, April 2012). For each sequence cluster, the closest relative is given (accession number in parentheses). Aligned pyroreads were inserted into the SILVA SSU Ref NR 111 guide tree using maximum parsimony criteria without changing the overall tree topology. The scale bar represents the number of substitutions for a unit branch length.
(TIFF)
