# Supplementary material for: Role of Diatoms in the Spatial-Temporal Distribution of Intracellular Nitrate in Intertidal Sediment
Source: PLoS One. 2013 Sep 4;8(9):e73257. doi: 10.1371/journal.pone.0073257 (PMC3762809; doi:10.1371/journal.pone.0073257)
Supplement: Table S1 — Number of operational taxonomic units (OTUs) observed and estimated in intertidal sediment and diatom mat samples from the German Wadden Sea. (DOC) [file pone.0073257.s001.doc]

**Table S1:** Number of operational taxonomic units (OTUs) observed and estimated in intertidal sediment and diatom mat samples from the German Wadden Sea

| **Sample** | **Sediment (Dec 2011)** | | | **Sediment (Jun 2011)** | | | **Diatom mat (Apr 2012)** | | |
| --- | --- | --- | --- | --- | --- | --- | --- | --- | --- |
| **Domain** | **Archaea** | **Bacteria** | **Eukarya** | **Archaea** | **Bacteria** | **Eukarya** | **Archaea** | **Bacteria** | **Eukarya** |
| **OTUs observed** | 13 | 314 | 137 | 27 | 392 | 357 | NA | 338 | 153 |
| **Richness S_Chao1_** | 13 | 250 | 136 | 27 | 419 | 357 | NA | 409 | 150 |

Phylotype richness was calculated using the bias-corrected estimator S_Chao1_ with a sequence similarity cutoff set at 97%.

Eukarya comprise Protista, Fungi, and Metazoa.
