# Supplementary figures and images for: Role of Diatoms in the Spatial-Temporal Distribution of Intracellular Nitrate in Intertidal Sediment
Source: PLoS One. 2013 Sep 4;8(9):e73257. doi: 10.1371/journal.pone.0073257 (PMC3762809; doi:10.1371/journal.pone.0073257)

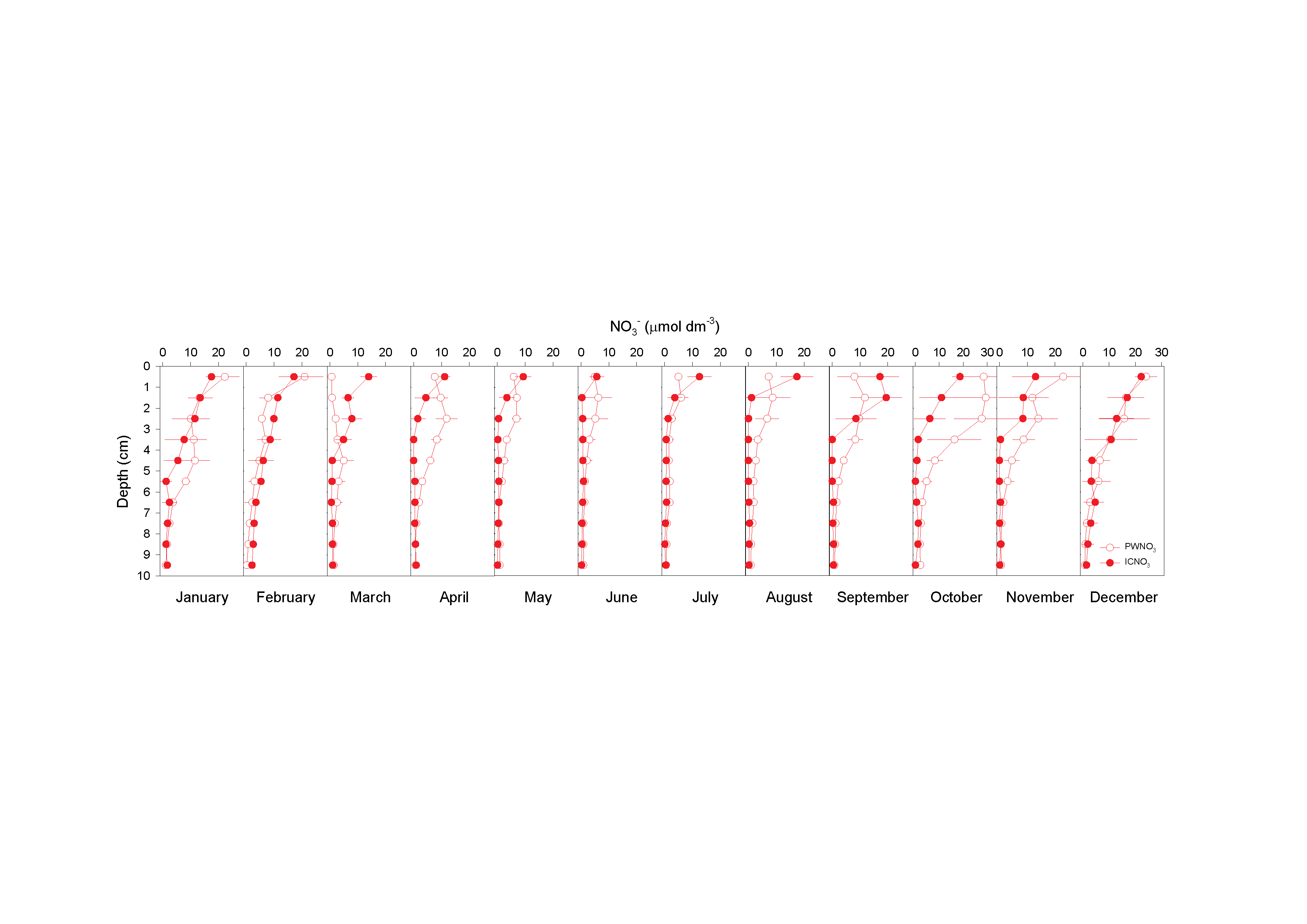

Supplement: Figure S1 — Seasonality of porewater nitrate (PWNO3) and intracellular nitrate (ICNO3) concentrations determined in monthly intervals in an intertidal flat of the German Wadden Sea. For each month, means ± s.d. of 3 replicate sediment cores are shown. (TIFF) [file pone.0073257.s002.tiff]

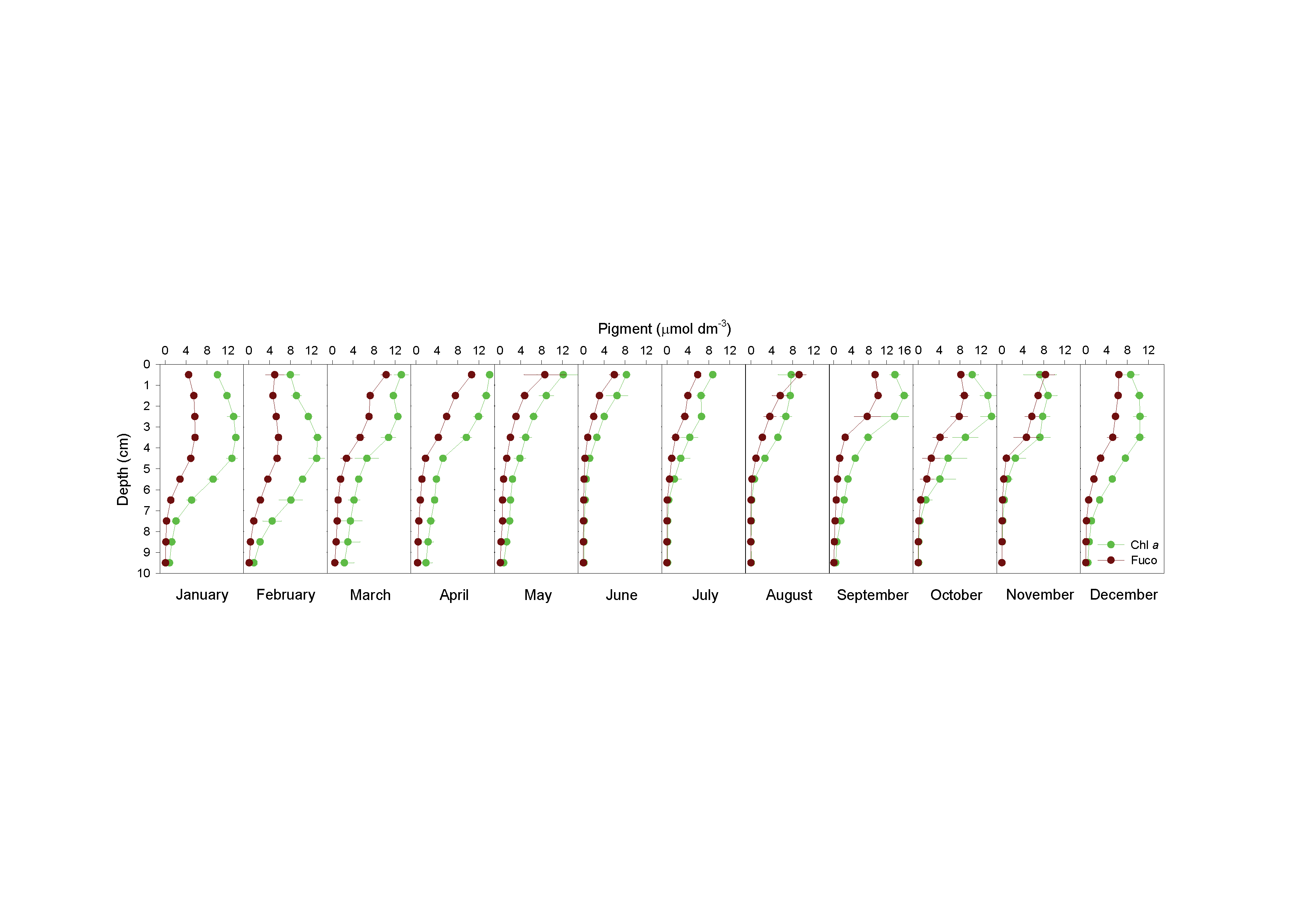

Supplement: Figure S2 — Seasonality of chlorophyll a (Chl a) and fucoxanthin (Fuco) concentrations determined in monthly intervals in an intertidal flat of the German Wadden Sea. For each month, means ± s.d. of 3 replicate sediment cores are shown. (TIFF) [file pone.0073257.s003.tiff]

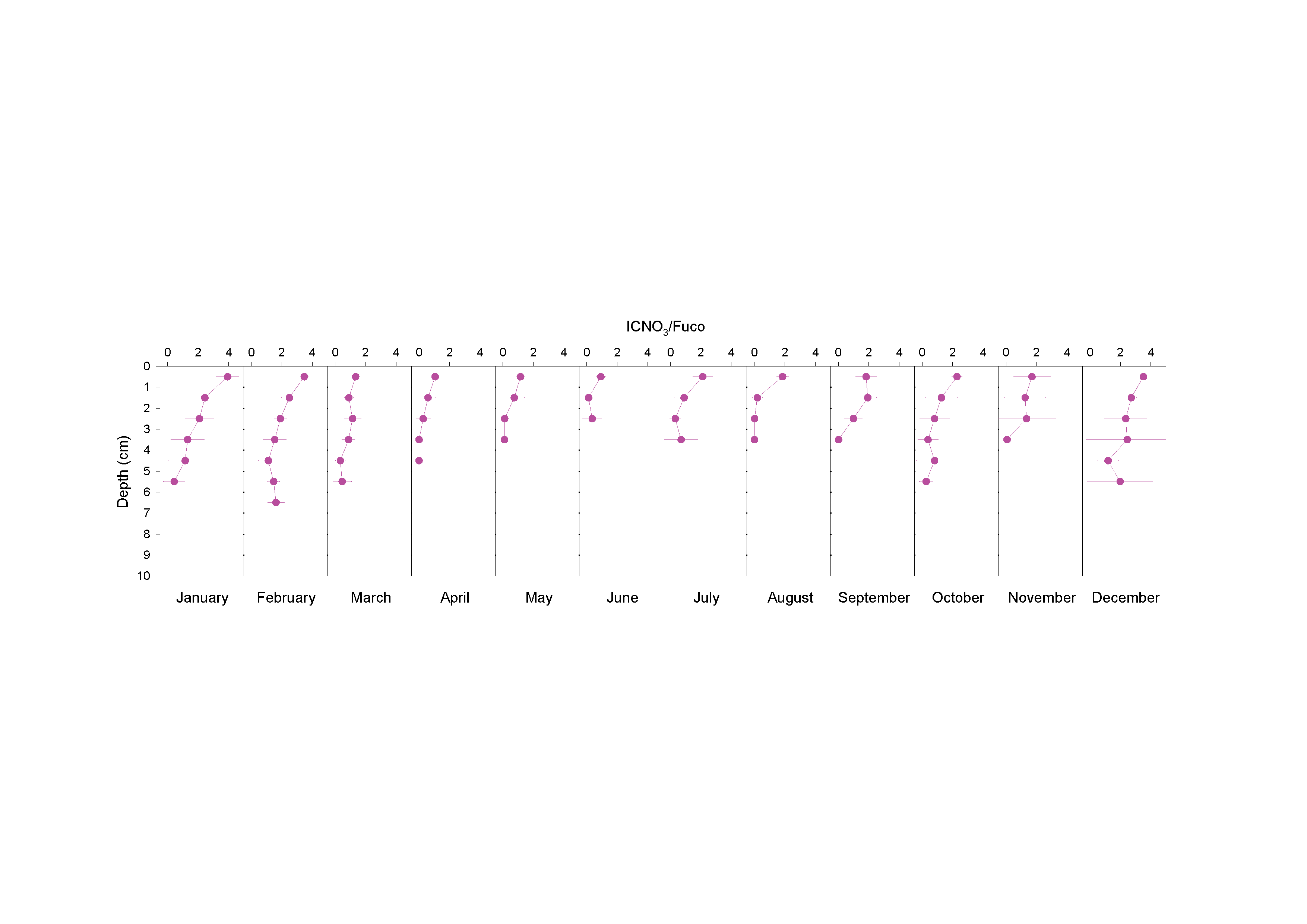

Supplement: Figure S4 — Seasonality of the molar intracellular-nitrate-to-fucoxanthin (ICNO3/Fuco) ratio in intertidal sediment sampled in monthly intervals in the German Wadden Sea. For each month, means ± s.d. of 3 replicate sediment cores are shown. (TIFF) [file pone.0073257.s005.tiff]

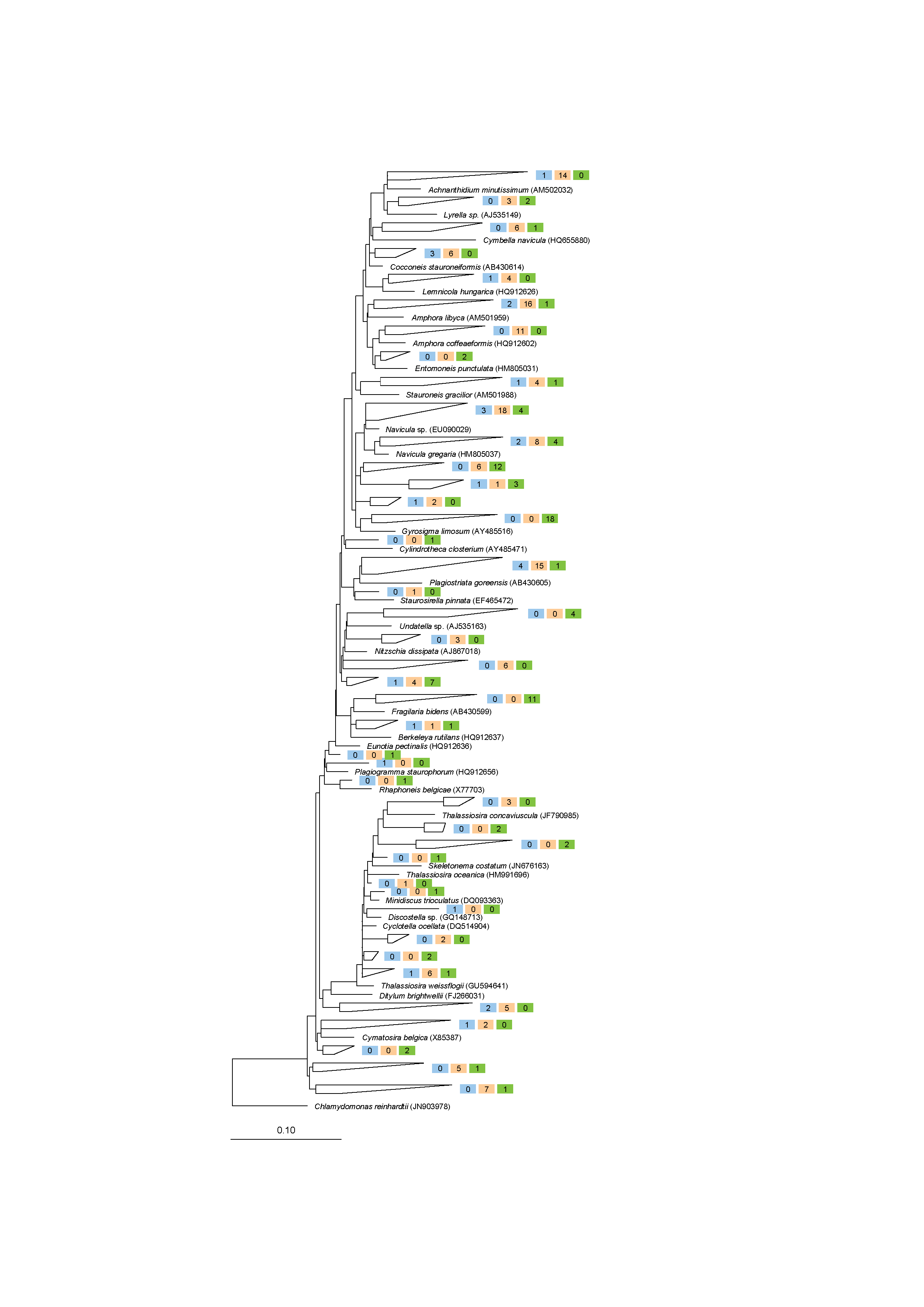

Supplement: Figure S5 — 18S rDNA-based phylogenetic reconstruction of Bacillariophyta in intertidal sediment and an intertidal diatom mat. The number of OTUs in each sequence cluster is given in the blue (Sediment, December 2011), red (Sediment, June 2011), and green boxes (Diatom mat, April 2012). For each sequence cluster, the closest relative is given (accession number in parentheses). Aligned pyroreads were inserted into the SILVA SSU Ref NR 111 guide tree using maximum parsimony criteria without changing the overall tree topology. The scale bar represents the number of substitutions for a unit branch length. (TIFF) [file pone.0073257.s006.tiff]
